# Targeting α-synuclein by PD03 AFFITOPE® and Anle138b rescues neurodegenerative pathology in a model of multiple system atrophy: clinical relevance

**DOI:** 10.1186/s40035-020-00217-y

**Published:** 2020-09-24

**Authors:** Miguel Lemos, Serena Venezia, Violetta Refolo, Antonio Heras-Garvin, Sabine Schmidhuber, Armin Giese, Andrei Leonov, Sergey Ryazanov, Christian Griesinger, Gergana Galabova, Guenther Staffler, Gregor Karl Wenning, Nadia Stefanova

**Affiliations:** 1 Division of Neurobiology, Department of Neurology, Innsbruck Medical University, 6020 Innsbruck, Austria; 2 AFFIRIS AG, Vienna, Austria; 3 Center for Neuropathology and Prion Research, Ludwig-Maximilians-University, Munich, Germany; 4 Max Planck Institute for Biophysical Chemistry, Göttingen, Germany; 5 Present Address: Origenis GmbH, Munich, Germany

**Keywords:** α-Synuclein, Immunotherapy, Oligomer modulation, Target engagement, Substantia nigra, Microglia

## Abstract

**Background:**

Misfolded oligomeric α-synuclein plays a pivotal role in the pathogenesis of α-synucleinopathies including Parkinson’s disease and multiple system atrophy, and its detection parallels activation of microglia and a loss of neurons in the substantia nigra pars compacta. Here we aimed to analyze the therapeutic efficacy of PD03, a new AFFITOPE® immunotherapy approach, either alone or in combination with Anle138b, in a PLP-α-syn mouse model.

**Methods:**

The PLP-α-syn mice were treated with PD03 immunotherapy, Anle138b, or a combination of two. Five months after study initiation, the mice underwent behavioral testing and were sacrificed for neuropathological analysis. The treatment groups were compared to the vehicle group with regard to motor performance, nigral neuronal loss, microglial activation and α-synuclein pathology.

**Results:**

The PLP-α-syn mice receiving the PD03 or Anle138b single therapy showed improvement of gait deficits and preservation of nigral dopaminergic neurons associated with the reduced α-synuclein oligomer levels and decreased microglial activation. The combined therapy with Anle138b and PD03 resulted in lower IgG binding in the brain as compared to the single immunotherapy with PD03.

**Conclusions:**

PD03 and Anle138b can selectively target oligomeric α-synuclein, resulting in attenuation of neurodegeneration in the PLP-α-syn mice. Both approaches are potential therapies that should be developed further for disease modification in α-synucleinopathies.

## Background

Parkinson’s disease (PD), dementia with Lewy bodies (DLB) and multiple system atrophy (MSA) are named α-synucleinopathies, because of the accumulation of misfolded α-synuclein (α-syn) in fibrillary intracellular inclusions, providing a diagnostic signature of these disorders. MSA is a fatal neurodegenerative disease clinically defined by a combination of autonomic dysfunction, atypical parkinsonism, cerebellar ataxia, and pyramidal signs [1]. The neuropathological hallmark of MSA is distinctive oligodendroglial cytoplasmic inclusions [2] containing misfolded fibrillary α-syn [3, 4]. α-Syn pathology has been suggested to be involved in the progression of the disease as it can spread throughout the brain, trigger neuroinflammation, and exert neurotoxicity [5–8]. Therefore, recent disease-modifying strategies are directed to clear and inhibit α-syn accumulation, and to limit the spread of pathological α-syn throughout the brain [9–12].

The PLP-α-syn transgenic mouse is a model of α-synucleinopathy that overexpresses human α-syn under the proteolipid protein (PLP) promoter in oligodendrocytes [13]. It recapitulates the glial cytoplasmic inclusion (GCI)-like pathology, as well as the selective neuronal loss and motor impairment [8, 13–15], replicating the Parkinson form of MSA (MSA-P). The oligomeric species of α-syn have been detected in the brains of PLP-α-syn mice at the time of initial microglial activation and neuronal loss in the substantia nigra pars compacta (SNc), supporting their role in the progression of the disease phenotype [8]. Therefore, the PLP-α-syn transgenic mice can be used to assess the efficacy of therapies targeting oligomeric α-syn species to ameliorate neurodegeneration. Recently, we have applied this model to study the disease-modifying effects of different α-syn-targeting strategies. The small molecule Anle138b through reduction of oligomeric α-syn in the brain rescued the motor phenotype and exerted neuroprotection in the PLP-α-syn mouse brain [12]. Reduction of C-terminal truncation by VX-765 (a caspase-1 inhibitor prodrug) decreased α-syn oligomers in the PLP-α-syn mice and consequently led to neuroprotection [16]. The molecular tweezer CLR01 successfully rescued nigral dopaminergic neurons in the PLP-α-syn model by reducing the level of high-molecular species of α-syn in the brain [17].

Immunotherapy targeting pathological α-syn is one of the attractive approaches for the treatment of PD, DLB, and MSA [11, 18–24]. Both active and passive immunization are under consideration. While passive immunization relies on the long-term application of selective antibodies [23], active immunization is based on the induction of long-lasting immune responses in the host by selective antigens [21].

In the current study, we set out to assess for the first time the efficacy of active immunotherapy with PD03, as well as the interactions of PD03 and Anle138b in a combined treatment, in the PLP-α-syn mouse model.

## Methods

### Study design and animals

Study I was aimed to assess the immunogenic response and the therapeutic potential of PD03. The age- and sex-matched PLP-α-syn mice received three subcutaneous injections of PD03 (200 μl each) (*n* = 12) or vehicle (*n* = 12) starting at 2 months of age (15 μg of the antigen peptide at week 0, 15 μg at week 2, and 75 μg for immune boosting at week 6 of the study). Immunogenicity was monitored by measuring specific antibody binding to human α-syn. Five months after initiation of the treatment, the mice were tested for motor deficits and thereafter sacrificed to examine the brain pathology. A group of wild-type age-, sex- and background-matched mice (*n* = 10) was used as the healthy control.

Study II was designed to replicate the efficacy evaluation of PD03 and Anle138b single therapies [12] in an independent experiment and to assess the interactions between the two treatments in combination. At 2 months of age, PLP-α-syn mice with matched age and sex were randomly assigned into four groups: PD03 group (*n* = 12), Anle138 group (*n* = 12), combined therapy (PD03 + Anle138b) group (*n* = 12), and vehicle group (*n* = 10). The immunotherapy followed the same scheme as in Study I. Anle138b was delivered with food pellets (ssniff Spezialdiäten GmbH, Soest, Germany) containing 0.6 g compound/kg of food as described previously [12]. Similarly, the immunogenicity was monitored by measuring specific antibody binding to human α-syn in the plasma, and 5 months after initiation of the treatment, the mice were tested for motor deficits and thereafter sacrificed to examine the brain pathology.

Mice were bred and maintained at the Animal Facility of the Medical University of Innsbruck at pathogen-free conditions in a temperature-controlled room with a 12 h/12 h light/dark cycle (lights on at 6 am) and free access to food and water. All in vivo experiments were approved by the Ethics Board at the Federal Ministry of Science and Research, Austria (permission BMWFW-66.011/0122-WF/V/3b/2014).

### PD03 vaccine formulation

Using the proprietary AFFITOME® technology, a short peptide termed PD03, which mimics part of the native sequence and structure of the human α-syn protein, was selected for further in vivo analysis. PD03 was synthesized at EMC microcollections (Tübingen, Germany) using conventional Fmoc chemistry, and then purified by HPLC to reach a purity grade of > 95%. To enable coupling of PD03 to carrier proteins, a cysteine was added to the N- or C-terminus. To chemically link the peptide to keyhole limpet hemocyanin (KLH), KLH was activated with the cross linker N-[γ-maleimidobutyryloxy]-succinimide ester (GMBS, Pierce) according to the manufacturer’s instructions. Prior to conjugation, the peptides were dissolved in 10% DMSO and then linked to activated KLH (50% of total volume) in 0.2 M Na-phosphate buffer (pH 6.8). The coupling efficiency was determined by Ellmann assay, which measures free SH groups, and by HPLC analysis before and after coupling. The PD03-KLH conjugates were adsorbed to aluminum hydroxide (Brenntag, Frederikssund, Denmark) as the adjuvant.

### Plasma sampling

Blood was collected by puncture of the facial vein at baseline before the initiation of immunotherapy (day 0), and on day 42 (week 6), day 71 (week 10), day 98 (week 14) and day 140 (week 20). Plasma was extracted after centrifugation and stored at − 80 °C until further analysis.

### Motor tests

#### DigiGait

Gait analysis was conducted using the Digigait System (Mouse Specifics, Quincy, MA, USA) to study gait changes. The mouse was placed on a motorized transparent treadmill belt. A video camera recorded the ventral side of the mouse, generating a video of the gait at a speed of 25 cm/s. The digital paw prints were analyzed with the DigiGait Software 9.0 (Mouse Specifics).

#### Challenging beam test

The motor coordination and balance was tested in a modified beam test and performed as previously described [25]. All animals were trained for five times in two consecutive days before the test. On the day of test, all animals were video-recorded for five beam crossings. All videos were then analyzed by an experimenter blinded to the treatment, and the number of slips per step (errors) was recorded.

### Enzyme-linked immunosorbent assay (ELISA)

To test antibody binding to human α-syn protein, 96-well Nunc MaxiSorp plates were coated with 1 μg/mL of recombinant human α-syn (rPeptide S-1001-2) diluted in 0.1 M NaHCO_3_ (pH 9.2). Then appropriate dilutions of plasma sample were added to the wells starting with a 1:100 dilution (dilution buffer: 1×PBS, 0.1% BSA, 0.1% Tween-20) and incubated for 1 h at 37 °C. Meanwhile, a standard antibody (LB509, Biolegend, #807702) was used as an internal control in each plate. After the incubation, 0.25 μg/mL of biotinylated anti-mouse IgG (H + L) (Southern Biotech, #1034–08; dilution 1:2000) was added for 1 h at 37 °C, followed by horseradish peroxidase-conjugated streptavidin (Roche, #1089153) for 30 min at 37 °C. Then the substrate ABTS (BioChemica, AppliChem) was added and incubated for 30 min at room temperature. The optical density was measured at 405 nm with a BioTek PowerWave 340 Plate Reader (Vermont, USA). The titers were defined as the plasma dilution factor at which 50% of the maximal optical density (ODmax) was reached.

### ELISA-based competition assay

ELISA plates were pre-coated with titrated amounts of different α-syn species, i. e. monomers (rPeptide) and fibrils (Proteos Inc), in descending concentrations from 100 μg/ml to 0.05 μg/ml (corresponding to 69 μM to 0.034 μM relative to monomeric α-syn). Then the PD03-specific antibodies purified from individual mice per group were pooled and added to the ELISA plates pre-coated with α-syn proteins. The α-syn species compete for the binding to the antibodies. IC50 values were calculated (using a non-linear regression analysis and a four-parameter logistic fit function provided by GraphPad PRISM® 5.04 software) to determine the concentration of monomeric or fibrillar α-syn needed to quench half of the ELISA signal.

### Tissue sampling

Mice were anesthetized with an overdose of thiopental and perfused transcardially with 20 ml of phosphate buffered saline (PBS, pH 7.4, Sigma-Aldrich, Vienna, Austria) for 5 min. Brains were rapidly removed and divided into hemispheres. One hemisphere was post-fixed in 4% paraformaldehyde (pH 7.4, Sigma-Aldrich, Vienna, Austria) overnight at 4 °C and then cryoprotected in 30% sucrose solution in PBS at 4 °C. Afterwards, the brains were frozen in 2-methylbutan (Merck, Darmstadt, Germany) and stored at − 80 °C until histological analysis. Midbrain of the other hemisphere was dissected, snap-frozen in liquid nitrogen and stored at − 80 °C for further biochemical analysis.

### Histological analysis

The fixed tissue was cut into 40-μm-thick sections on a freezing microtome (Leica, Nussloch, Germany) in five series. Immunohistochemistry was performed according to a standard protocol for free-floating sections using the following antibodies: monoclonal mouse anti-tyrosine hydroxylase (TH; 1:700, Sigma-Aldrich, Vienna, Austria), anti-phospho-synuclein (pS129, 1:1000, Abcam, UK), anti-Iba1 (1:300, Abcam, UK) and anti-CD68 (1:200, Serotech, Oxford, UK). The sections were then incubated with the appropriate biotinylated secondary antibody followed by Vectastain ABC reagent (Vector Laboratories, Burlingame, CA, USA) and 3,3′-diaminobenzidine (Sigma-Aldrich, Vienna, Austria) to visualize the immunohistochemical binding sites. The stained sections were mounted on slides, dehydrated and coverslipped with Entellan (Merck, Darmstadt, Germany).

IgG binding in the brain parenchyma was assessed by incubating brain sections with goat anti-mouse IgG conjugated to Alexa 488 (1:1000, Life Technologies). The sections were mounted on slides and coverslipped with Fluoromount-G (Southern Biotech).

### Image analysis

Sections were observed under a Nikon E-800 microscope equipped with Nikon digital camera DXM 1200 and the images were analyzed with the Stereoinvestigator Software (MicroBrightField Europe e.K., Magdeburg, Germany) as described previously [8]. The image analysis was performed in a blinded manner. The numbers of TH-positive neurons and neurons with cresyl-violet staining in the SNc were assessed stereologically by the optical fractionator. The density of pS129-positive inclusions was assessed in SNc with the meander scan using the Stereoinvestigator software. The meander scan method was also used to determine the density of Iba1-immunoreactive microglia in SNc. The Iba1-positive cells were further analyzed for the density of homeostatic (type A) and activated (type B, C, D) microglia as previously described [8, 26]. CD68-immunoreactivity was assessed by quantifying the density of immunopositive clusters and applying a pathological rating scale (+, a single cluster; ++, 2–5 clusters; +++, 6–10 clusters; ++++, > 10 clusters). The binding of IgGs in the brain parenchyma was measured by Image J in pictures taken at 20× objective with constant light and exposure settings on a Leica DMi8A microscope installed with LMC imaging software. In ImageJ, each image was converted to 8-bit, and a threshold was set to exclude vessels from the measurements and assess only fluorescence from IgG binding in the parenchyma. Finally, the percentage of fluorescent area was measured and used for the statistical analysis.

### Alpha-synuclein solubility fractionation and western blot analysis

The snap-frozen tissue was homogenized in TX extraction buffer (150 mM NaCl, 50 mM Tris pH 7.6, 1% Triton-X-100, 2 mM EDTA) containing protease and phosphatase inhibitors. The lysate was sonicated and centrifuged (120,000 × g for 60 min at 4 °C) and the supernatant was collected as the TX-soluble fraction. The pellet was washed three times with 1 M PBS/1% TX, centrifuged (13,000 × g for 15 min), re-suspended in SDS extraction buffer (150 mM NaCl, 50 mM Tris pH 7.6, 1% TX, 0.5% Na deoxycholate, 1% SDS), sonicated, and left on ice for 30 min followed by centrifugation at 120,000 × g for 60 min at 4 °C. The supernatant was collected as SDS-soluble fraction. The pellet was washed three times with 1 M PBS/1% SDS, centrifuged (13,000 × g for 15 min), dissolved in 8 M urea/SDS (5%) solution, mixed and boiled for 5 min, centrifuged at 120,000 × g for 60 min at 18 °C, and the supernatant was collected as the urea-soluble fraction. Protein levels were measured with the BCA method and 30 μg of protein samples were loaded on the NuPage 4–12% Bis-Tris gel (Invitrogen). Protein separation was done using the Novex Mini-Gel chambers (Life Technologies) by gel electrophoresis for 1 h at 200 V. Proteins were transferred to the Invitrolon™ PVDF membrane (Invitrogen) for 90 min at 30 V. Ponceau S solution (Sigma) was used to confirm the protein transfer. The membranes were blocked in 2% Amersham ECL Prime Blocking Agent (GE Healthcare) for 1 h. Primary antibodies included anti-α-synuclein (Genetex, GTX21904, monoclonal, 1:5000) and anti-β-III-tubulin (Milipore, AB9354, polyclonal, 1:10000) as a loading control. Imaging was performed with the Fusion FX system (Vilber Lourmat, Marne La Vallée, France), and densitometric analysis was carried out using FUSION CAPT V16.09b software and ImageJ.

### Statistical analyses

All statistical analyses were conducted using GraphPad Prism 8 (Graphpad Software, San Diego, CA). Data are presented as mean ± SEM and were analyzed with one-way analysis of variance (ANOVA) with *post-hoc* Bonferroni test unless indicated otherwise. Linear regression analysis was applied to correlate different parameters. *P* < 0.05 was considered as statistically significant.

## Results

### PD03 is immunogenic and ameliorates motor symptoms and nigral degeneration in PLP-α-syn mice

**Fig. 1:**
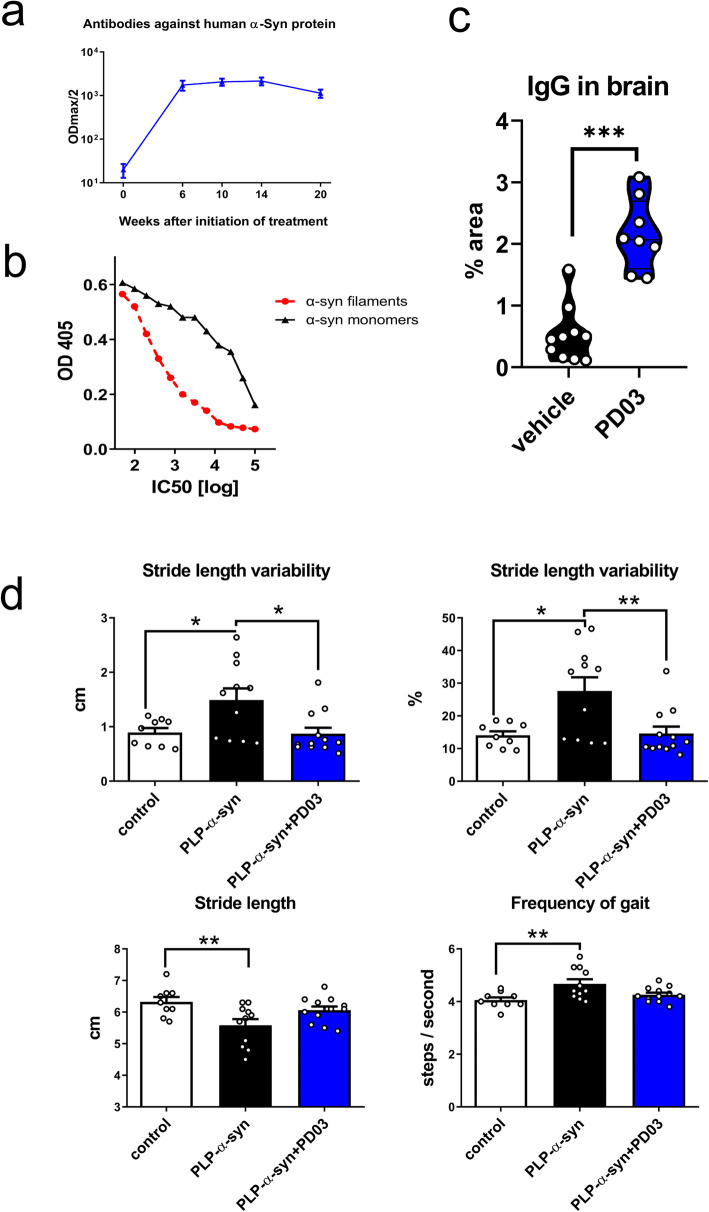
Immunogenicity of PD03 in PLP-α-syn mice, antibody binding in the brain, and gait analysis. **a** The plasma levels of antibodies binding to human α-syn after PD03 immunotherapy reached a high level at week 6 and remained stable thereafter. **b** ELISA-based competition assay demonstrating the competitive binding of PD03 antibodies to α-syn monomers and fibrils. **c** Percentage of area positive for IgG binding in the brains of vehicle- and PD03-treated PLP-α-syn mice (two-tailed *t*-test, *n*_vehicle_ = 8, *n*_PD03_ = 10, *t*_16_ = 6.742, ***P < 0.0001). **d** DigiGait analysis of wild-type controls, and PLP-α-syn mice receiving vehicle or PD03. Data were analyzed with one-way ANOVA followed by Bonferroni’s *post-hoc* test (*P < 0.05), **P < 0.01

In study I, PLP-α-syn mice were treated with PD03 conjugated to KLH as the carrier to target α-syn pathology in MSA mice. Two out of 12 transgenic mice died in the vehicle-treated PLP-α-syn group, while no death occurred in the PD03-treated PLP-α-syn mice and the healthy control group. There was no significant difference among the survival curves (log-rank test: *χ*^2^ = 3.83, df = 2, *P* = 0.1473). The PLP-α-syn mice immunized with PD03 had high titers of antibodies reactive to human α-syn, while the antibodies were not detectable before the initiation of the treatment (day 0) or in the vehicle-treated mice during the entire experiment (Fig. 1a). To test the binding preference of PD03-induced antibodies to different α-syn species, we used an ELISA-based competition assay. α-Syn fibrils competed for binding of PD03 antibodies (purified from mice after the third immunization) at a concentration of 0.46 μg/ml (IC50), whereas a concentration of 36.4 μg/ml was needed for α-syn monomers to achieve the same quenching effect. These data demonstrated that the PD03-induced antibodies preferentially bind to higher-order α-syn species (Fig. 1b). Post-mortem analysis of the brain showed significantly increased IgG binding in the brain parenchyma in the PD03-immunized mice as compared to the vehicle-treated animals (Fig. 1c), indicating that the PD03 antibodies could cross the blood-brain barrier and accumulate in the brain.

Nineteen weeks after the first injection, mouse motor performance was tested by Digigait analysis. After Bonferroni’s multiple comparison test, the PLP-α-syn mice showed significant shortening of the stride length (*t* = 3.201, *P* < 0.01), increase of the gait frequency (*t* = 3.329, *P* < 0.01), and increase of the stride length variability (CV (cm), *t* = 2.7, *P* < 0.05 and CV (%), *t* = 3.189, *P* < 0.05) which were consistent with previous observations [8, 14]. The PLP-α-syn mice receiving PD03 showed stride length variability comparable to the healthy wild-type level but significantly lower than the vehicle-treated mice (Fig. 1d). Further, PD03 treatment seemed to reverse the shortening of the stride length and the increased stride frequency of PLP-α-syn mice compared to the vehicle-treated animals, but the difference was not statistically significant (Fig. 1d).

**Fig. 2:**
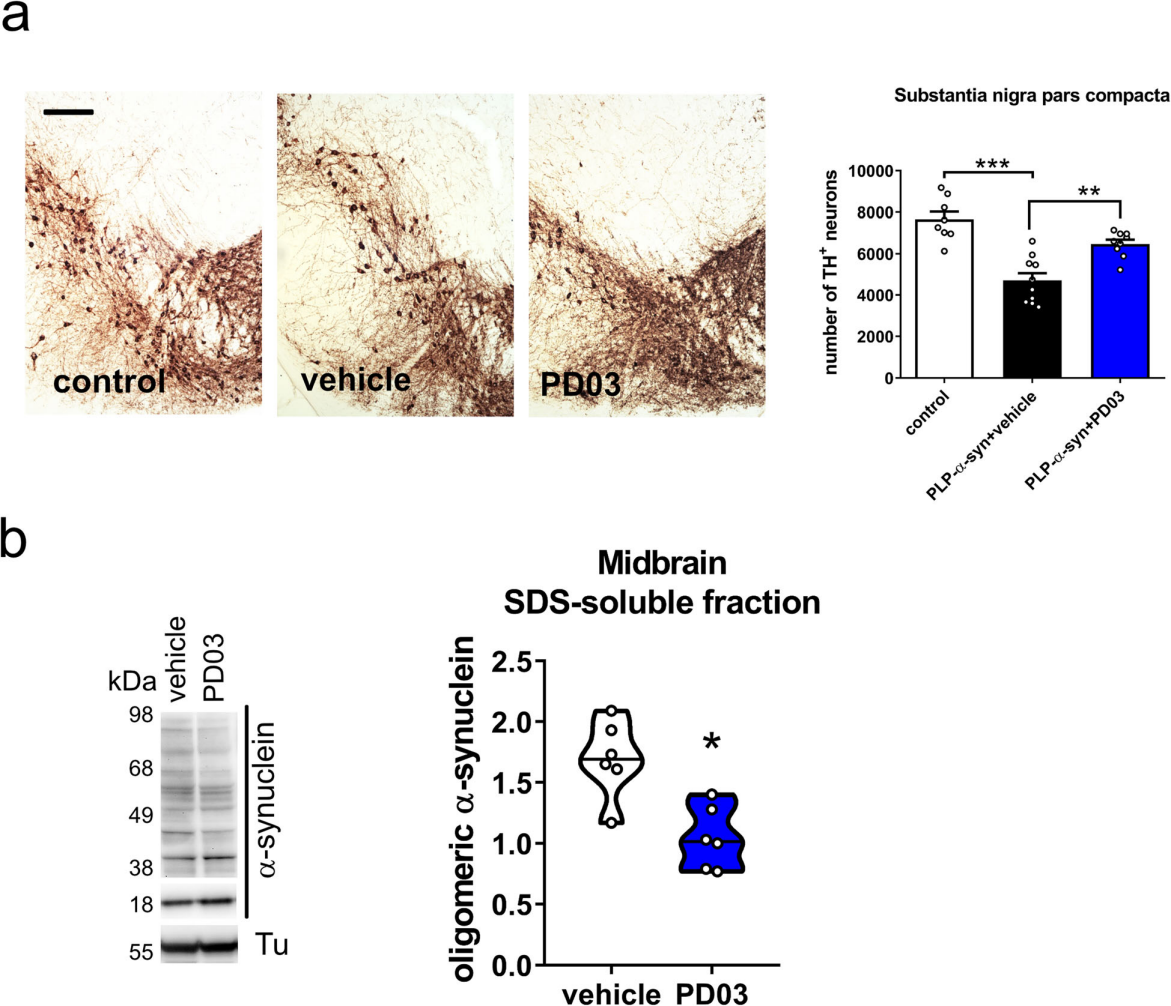
Nigral neuronal loss and abundance of α-syn species (monomers and oligomers) in the midbrain of wild-type controls, and PLP-α-syn mice receiving vehicle or PD03 immunotherapy. **a** TH immunohistochemistry to define the number of dopaminergic neurons in SNc. Data are presented as mean ± SEM. One-way ANOVA with *post-hoc* Bonferroni test, **P < 0.01. ***P < 0.001. Scale bar, 50 μm. **b** Densitometric analysis of oligomeric α-synuclein in the SDS-soluble fraction. Values represent relative intensity versus control protein (β-III-tubulin). Mann-Whitney test, *P < 0.05

Previous reports have shown a loss of dopaminergic neurons in the SNc of PLP-α-syn mice after the age of 4 months [7, 8, 14, 15]. Consistently, there was a significant loss of TH-positive neurons in the vehicle-treated PLP-α-syn mice as compared to the wild-type controls (Bonferroni’s multiple comparisons test, *t* = 6.531, *P* < 0.0001, Fig. 2a). PD03 immunotherapy effectively rescued the dopaminergic neuron loss in the SNc as compared to the vehicle-treated PLP-α-syn mice (Bonferroni’s multiple comparisons test, *t* = 4.039, *P* < 0.01, Fig. 2a). Further, we identified a correlation between the degree of nigral neurodegeneration and the severity of gait deficit (stride length: *R*^2^ = 0.4165, *P* = 0.0005; frequency of gait: *R*^2^ = 0.4684, *P* = 0.0002; stride length variability (cm): *R*^2^ = 0.3472, *P* = 0.0019; stride length variability (%): *R*^2^ = 0.4559, *P* = 0.0002).

### PD03 immunization induces significant reduction of oligomeric forms of α-syn in the midbrain of PLP-α-syn mice

Midbrain samples were homogenized and the TX-soluble, SDS-soluble, and urea-soluble proteins were sequentially extracted. The levels of TX-soluble and SDS-soluble monomers showed no significant differences between the vehicle and PD03 groups (Mann-Whitney test, median ± SD, TX-soluble fraction: vehicle 1.29 ± 0.48, *n* = 6; PD03 1.085 ± 0.17, *n* = 6; *P* = 0.6991; SDS-soluble fraction: vehicle 1.16 ± 0.56, *n* = 6; PD03 0.84 ± 0.67, *n* = 6; *P* = 0.6991). However, the oligomeric α-syn expression in the SDS-soluble fraction was decreased after PD03 immunotherapy in the PLP-α-syn mice (Fig. 2b). We found a significant correlation between the nigral neuronal loss and the level of SDS-soluble α-syn oligomers in the midbrain of PLP-α-syn mice (*R*^2^ = 0.6464, *P* = 0.0051). In the urea-fraction, only monomeric α-syn was measurable (Mann-Whitney test, median ± SD, vehicle 0.76 ± 0.18, *n* = 6; PD03 0.82 ± 0.09, *n* = 6; *P* = 0.0931), either due to the SDS-solubility of the α-syn oligomers in PLP-α-syn mouse brain as in human MSA [27, 28], or because the levels were too low to quantify.

### PD03 immunotherapy reduces microglial activation in PLP-α-syn mice

**Fig. 3:**
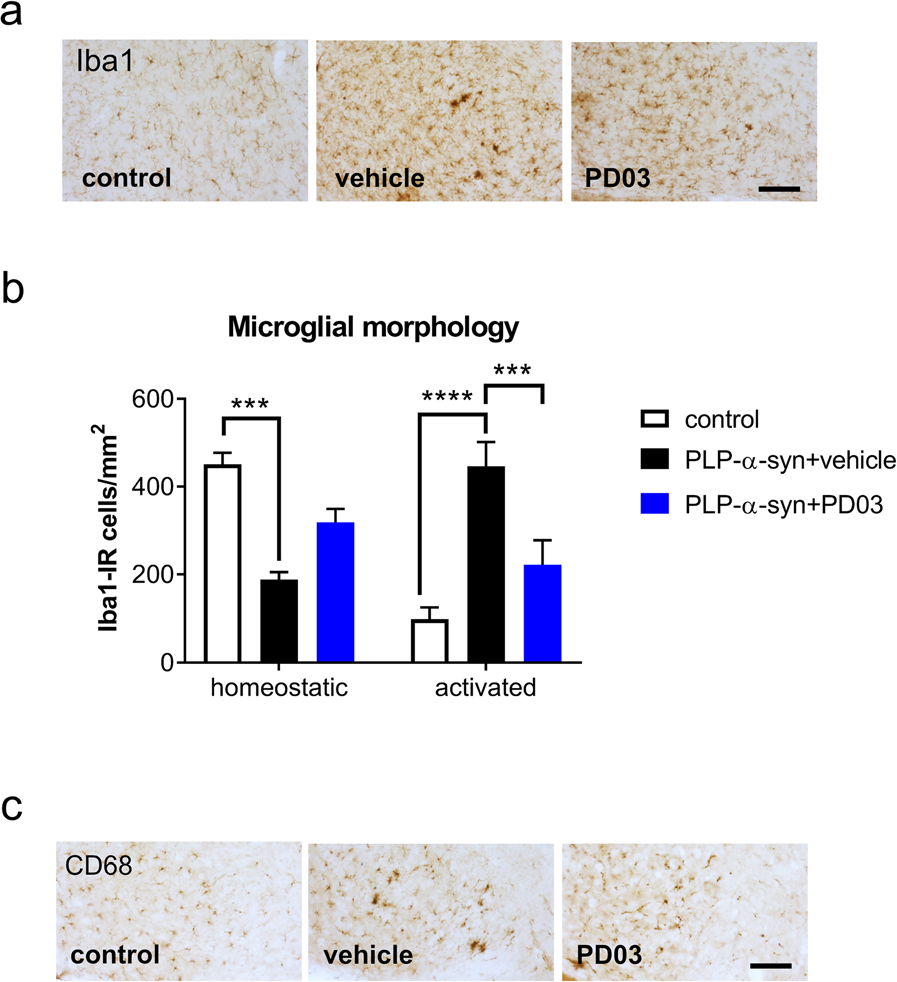
Microglial activation in the SNc after PD03 immunotherapy. **a** Iba-1 immunohistochemistry in SNc. **b** Densities of homeostatic and activated microglia. Data are presented as mean ± SEM. Two-way ANOVA with *post-hoc* Bonferroni test, ***P < 0.001, ****P < 0.0001. **c** CD68 immunohistochemistry in SNc. Scale bars, 100 μm

The total density of Iba1-positive cells in the SNc did not differ between the vehicle and PD03 groups (one-way ANOVA, *F*_2,12_ = 2.19, *P* = 0.1546). However, the morphological profiles of activated microglia in the SNc showed significant group differences. The PLP-α-syn mice receiving vehicle showed a shift towards activated microglia as compared to the control wild-type mice. PD03 treatment resulted in a significantly lower density of activated microglia and a shift towards the homeostatic profile (Fig. 3a, b). This finding was supported by CD68 immunostaining, which showed reduction of phagocytic CD68-positive clusters in the PD03-treated PLP-α-syn mice (Fig. 3c).

### Efficacy and target validation of PD03 and Anle138b in PLP-α-syn mice

**Fig. 4:**
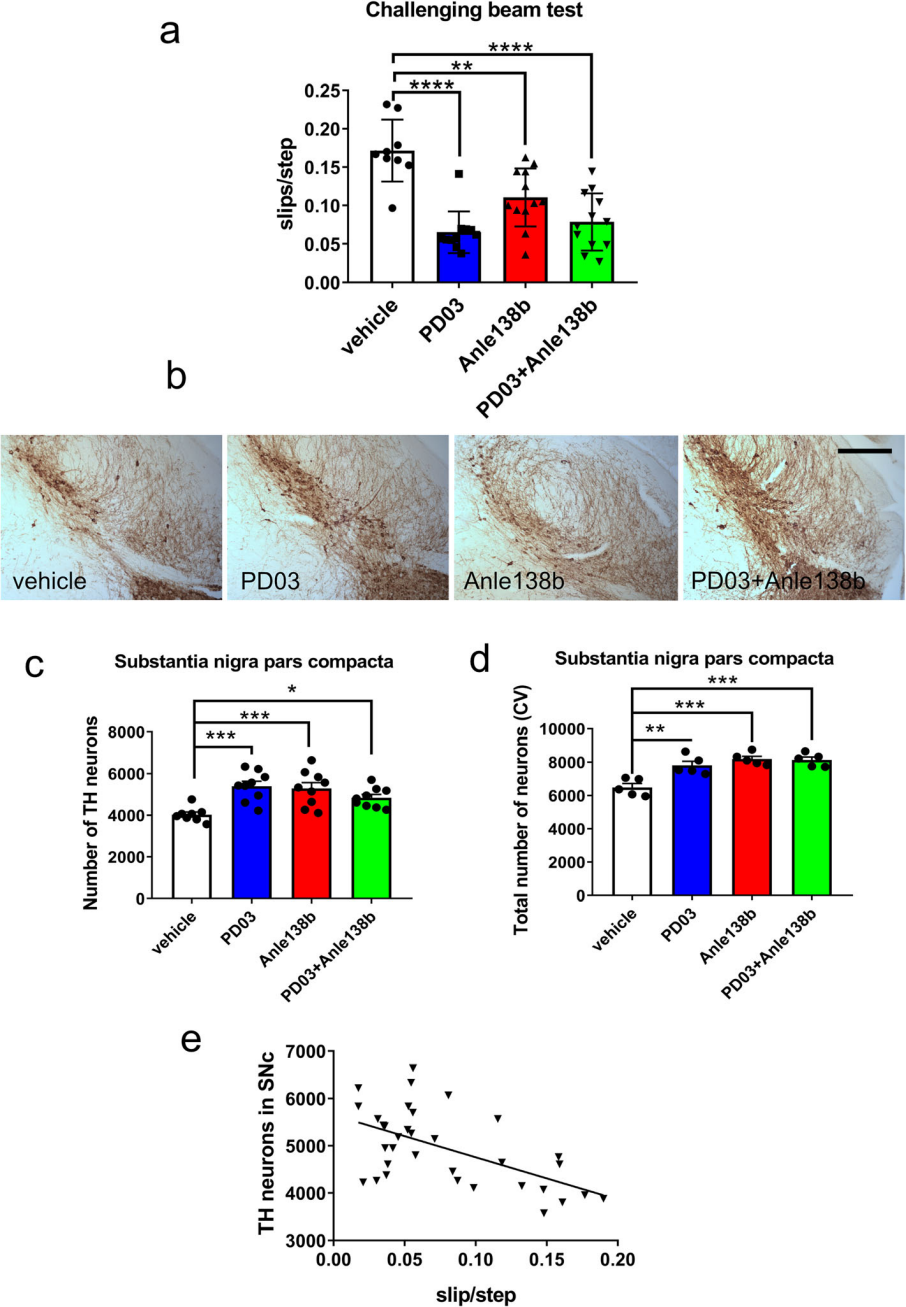
Effect of single or combined treatment with Anle 138b and PD03 on the motor disability and nigral neurodegeneration in PLP-α-syn mice. **a** Challenging beam test of PLP-α-syn mice receiving vehicle, PD03, Anle138b or combined PD03 + Anle138b treatment. One-way ANOVA followed by Bonferroni’s *post-hoc* test (**P < 0.01; ****P < 0.0001). **b** Immunohistochemistry of TH to identify dopaminergic neurons in the SNc of PLP-α-syn mice. **c** Number of TH-immunoreactive neurons in the SNc of PLP-α-syn mice receiving vehicle, PD03, Anle138b or combined PD03 + Anle138b treatment. One-way ANOVA followed by Bonferroni’s *post-hoc* test (***P < 0.001, *P < 0.05). **d** Total number of neurons in the SNc of PLP-α-syn mice counted in cresyl violet (CV) staining. One-way ANOVA followed by Bonferroni’s *post-hoc* test (***P < 0.001, **P < 0.01). **e** Linear regression analysis of correlations between dopaminergic neurons in the SNc and motor disability in the challenging beam test (*R*^2^ = 0.333, *P =* 0.0004)

To confirm the efficacy of PD03 and Anle138b [12], a second cohort of PLP-α-syn mice was treated with vehicle, PD03, Anle138b, and combined PD03 + Anle138b. To evaluate motor coordination and balance, all the PLP-α-syn mice underwent the challenging beam test, a highly sensitive test for varying degrees of nigrostriatal dopaminergic dysfunction in genetic mouse models [29]. The mice treated with PD03 only, Anle138b only, or combined therapy of PD03 + Anle138b showed a significant decrease in the number of slips per step as compared to the vehicle-treated mice (Fig. 4a).

TH immunohistochemistry confirmed the rescue of dopaminergic neurons in the SNc in PLP-α-syn mice treated with Anle138b, PD03 alone or the combination of PD03 and Anle138b as compared to the vehicle-treated mice (Fig. 4b, c). To provide evidence that this result reflected actual neuronal rescue rather than the changes of TH expression, we further conducted cresyl-violet staining to estimate the total neuron number in the SNc. The results confirmed the neuroprotective efficacy of Anle138b and PD03, either alone or in combination (Fig. 4d). Importantly, the higher number of dopaminergic neurons in the SNc was significantly correlated with the improved performance (less errors) of the PLP-α-syn mice in the challenging beam test (Fig. 4e).

**Fig. 5:**
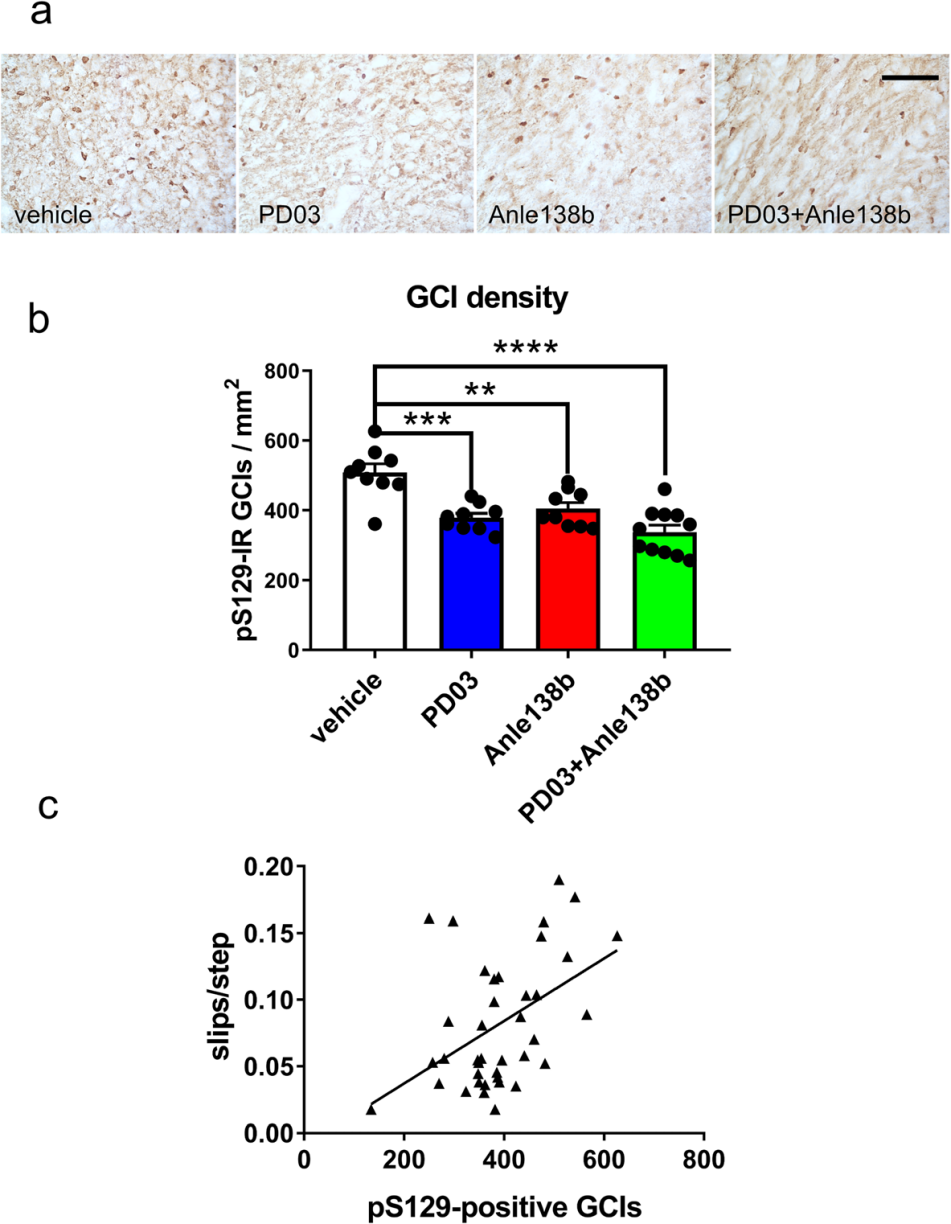
Effect of single or combined treatment with Anle 138b and PD03 on the GCI pathology in PLP-α-syn mice. **a** Immunohistochemistry of pS129 to identify GCI in the brains of PLP-α-syn mice. **b** GCI density in the SNc of PLP-α-syn mice receiving vehicle, PD03, Anle138b or combined PD03 + Anle138b treatment. ****P < 0.0001, ***P < 0.001, **P < 0.01, one-way ANOVA followed by Bonferroni’s *post-hoc* test. **c** Linear regression analysis of correlations between GCI density in the SNc and motor disability in the challenging beam test of PLP-α-syn mice (*R*^2^ = 0.216, *P =* 0.003)

In addition, pS129 immunohistochemistry showed a reduction in the number of GCIs in all treatment groups as compared to controls receiving vehicle (Fig. 5a, b). Interestingly, a correlation between GCI accumulation and motor deficits was observed, where a higher number of GCIs in the SNc significantly correlated with a higher number of errors in the challenging beam test (Fig. 5c).

**Fig. 6:**
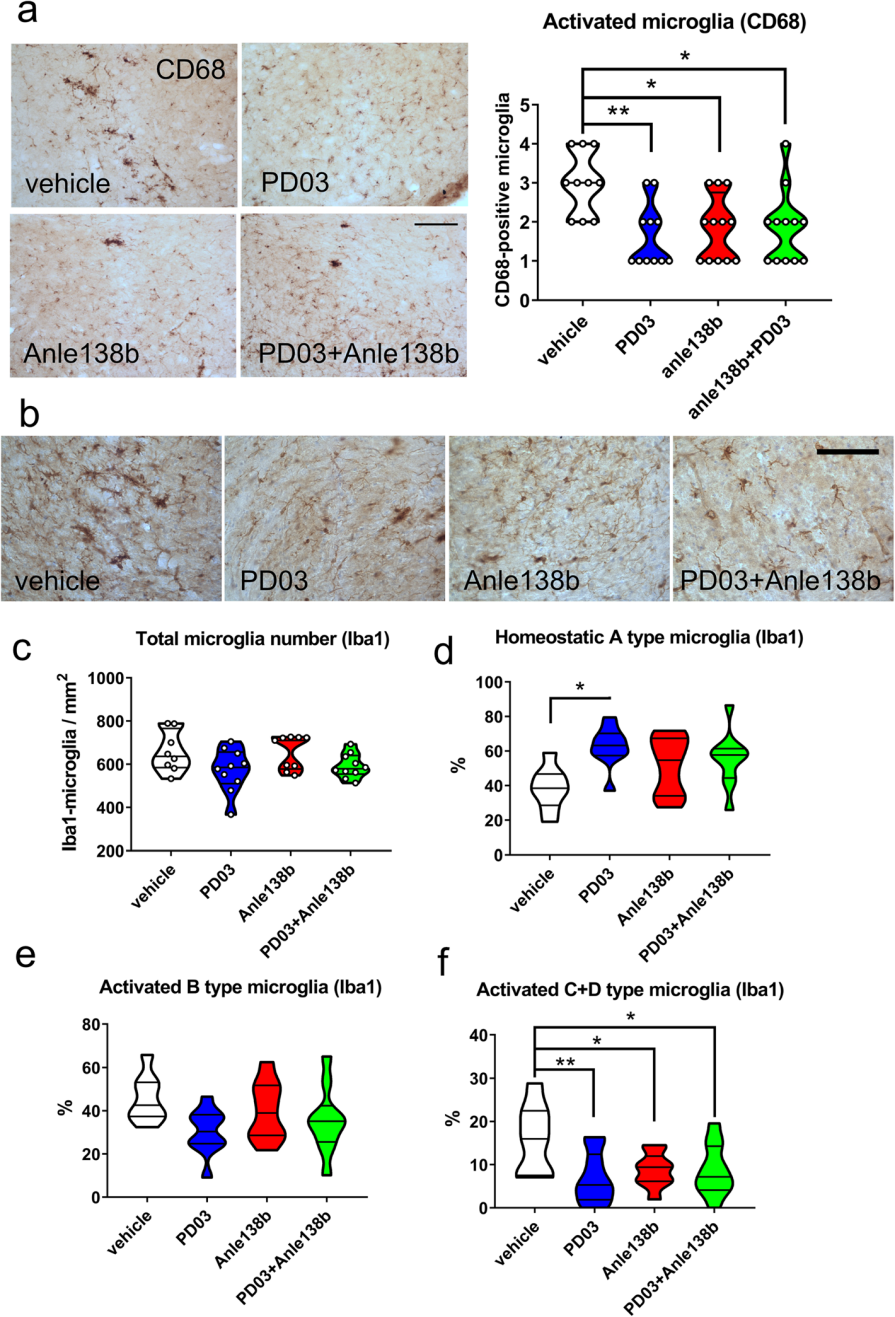
Effects of single or combined treatment with Anle 138b and PD03 on microglial activation in SNc. **a** CD68 immunohistochemistry in SNc (left) and a violin plot of the density of activated microglia in CD68 immunohistochemistry in SNc (right). *P < 0.05, **P < 0.01, one-way ANOVA with *post-hoc* Bonferroni test. **b** Iba-1 immunohistochemistry in SNc. Scale bars, 100 μm. **c** Total number of Iba-1-immunoreactive microglia in the SNc of PLP-α-syn mice receiving different treatment. **d** Percentage of homeostatic (type A) Iba-1-immunoreactive microglia in the SNc of PLP-α-syn mice receiving different treatment. **e** Percentage of B-type activated Iba-1-immunoreactive microglia in the SNc of PLP-α-syn mice receiving different treatments. **f** Percentage of activated (C-D type) Iba-1-immunoreactive microglia in the SNc of PLP-α-syn mice receiving different treatments. *P < 0.05, **P < 0.01, one-way ANOVA with *post-hoc* Bonferroni test

It has been indicated that the microglial activation and the phagocytic activity are associated with the α-syn pathology in the brains of PLP-α-syn mice [8]. To analyze the effects of the therapies targeting α-syn on microglial responses, CD68-positive clusters of activated microglia were assessed in the SN of PLP-α-syn mice in all experimental groups. Results showed that the phagocytic microglial activation in the SN of PLP-α-syn mice was decreased following treatment with Anle138b and PD03, either alone or in combination (Fig. 6a). In parallel, the number and the morphological phenotypes of microglia were assessed by Iba1 immunohistochemistry (Fig. 6b). There was no significant difference in the total number of microglia in the SNc among the four experimental groups (Fig. 6c). The percentage of homeostatic (A type) microglia was significantly increased in the PD03 group (Fig. 6d). The B-type activation of microglia was not affected by any of the treatments (Fig. 6e), but the activated microglia with shorter processes and amoeboid soma, i.e. phagocytic cells (C and D types [8, 26]), were significantly reduced in all therapy groups versus the vehicle treated group (Fig. 6f). The changes in the microglial phagocytic response correlated with the amelioration of the α-syn inclusion pathology in the PLP-α-syn mice (linear regression analysis, *R*^2^ = 0.14, *P* = 0.04).

**Fig. 7:**
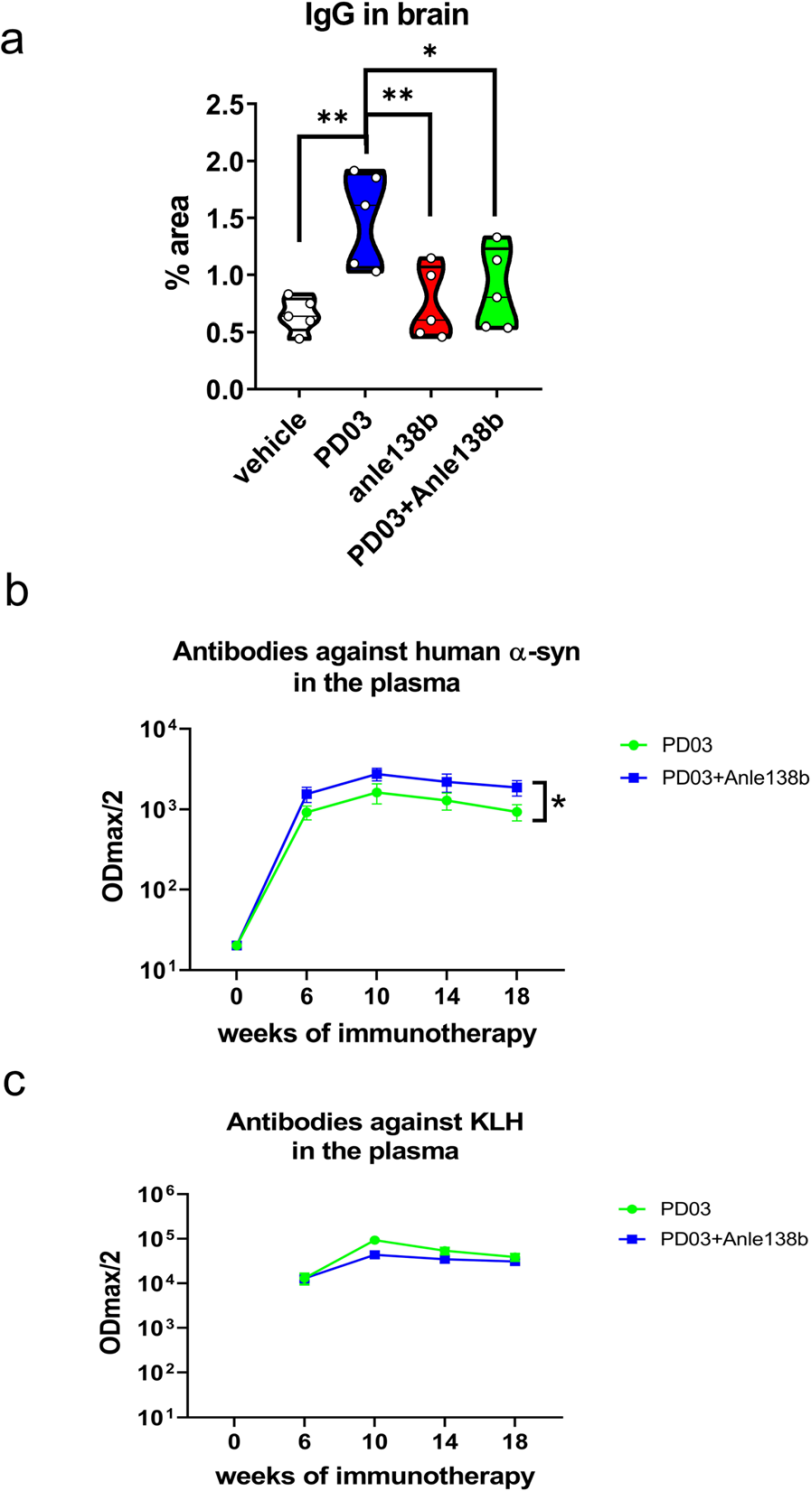
Effect of single or combined treatment with Anle 138b and PD03 on IgG binding in the brain and on the plasma antibody titers in PLP-α-syn mice. **a** IgG binding analysis. *P < 0.05; **P < 0.01, one-way ANOVA with *post-hoc* Tukey’s test. **b** Kinetics of plasma titers of anti-α-syn antibodies. Area under ROC curve analysis (*P = 0.0433). **c** Kinetics of plasma titers of anti-KLH antibodies. Area under ROC curve analysis (*P =* 0.2482)

Finally, we examined whether α-syn oligomer modulation by Anle138b would interfere with the levels of antibodies in the brain parenchyma after treatment with PD03. It has been reported and repeatedly confirmed that Anle138b acts as an oligomer modulator for α-syn [12, 30, 31]. On the other hand, in Study I we identified higher binding of PD03-induced antibodies to higher-order α-syn species (Fig. 1c). Intriguingly, when Anle138b and PD03 were combined, we detected reduced antibody binding in the brain parenchyma as compared to that after PD03 treatment alone (Fig. 7a), suggesting that the reduced α-syn pathology via Anle138b-induced oligomer modulation results in reduced accumulation of antibodies in the brain. This was further supported by the measurement of free anti-α-syn antibody titers in the plasma of immunized mice. Antibody titers against human α-syn were stably present in the plasma of mice treated with PD03 either alone or in combination with Anle138b. Interestingly, the plasma titers of free anti-α-syn antibodies were higher in mice treated with PD03 + Anle138b as compared to that with PD03 alone through area under the Receiver Operating Characteristics Curve analysis (Fig. 7b). Yet, the same groups showed no difference in the plasma titers of antibodies to the carrier KLH (Fig. 7c).

## Discussion

Our current findings showed that the active immunotherapy with PD03 and the treatment with the oligomer modulator Anle138b reliably ameliorated α-syn pathology in the midbrain of PLP-α-syn mice, associated with neuroprotection of nigral dopaminergic neurons, resulting in attenuation of the motor deficits. Next, we showed that the anti-α-syn antibodies elicited by PD03 immunotherapy preferentially targeted higher-order α-syn species and the modification of α-syn oligomers through Anle138b resulted in reduced accumulation of IgG in the brain parenchyma in a combined therapy approach. These findings support the specific target engagement by both approaches.

In the current study, we replicated in an independent experiment the efficacy of Anle138b in the PLP-α-syn mice as reported previously [12] and confirmed the feasibility of the oligomer modulation approach for the treatment of α-synucleinopathies. Immunotherapies targeting α-syn pathogenic species are currently tested as an alternative approach for disease modification in PD, DLB and MSA [11, 21, 32]. Passive immunization with various selective antibodies has been reported to have differential and region-specific effects on α-syn pathology linked to mild motor improvement [23, 33–37]. The earliest attempts of active immunization with α-syn were reported in 2005 by Masliah et al. [18], who showed decreased accumulation of aggregated α-syn associated with reduced neurodegeneration following active immunization with α-syn in transgenic mice. The next steps in this direction involved the development of immunotherapies with higher safety and minimized autoimmune side effects based on the use of short peptides (AFFITOPEs®) that mimic specific antigen epitopes [19]. Initial studies in PD, DLB, and MSA mouse models demonstrated the efficacy of another α-syn AFFITOPE®, PD01, which triggered specific antibody generation with CNS penetration and lowered α-syn aggregates and oligomers, leading to neuroprotection and improvement of locomotor behavior [38, 39]. The first-in-human application of PD01 in PD patients has demonstrated safety and immunogenicity [40]. The recent findings of variable α-syn filaments in different α-synucleinpathies [28, 41] suggest that vaccines may show different efficacies in these disorders depending on the epitope binding. Therefore, the development of different vaccines is of high clinical relevance. PD01 and PD03 AFFITOPEs differ in peptide length (7 and 9 amino acids, respectively) and amino acid composition (two versus three exchanges compared to the native sequence, respectively). Antibodies induced by PD01 and PD03 AFFITOPEs bind to slightly different but overlapping epitopes present in the C-terminal region of human α-syn, but they both preferentially bind oligomeric and aggregated forms of α-syn.

In the current study we assessed for the first time the in vivo effects of the new AFFITOPE® PD03 in a mouse model of α-synucleinopathy. In two independent experiments, we demonstrated that PD03 was highly immunogenic in PLP-α-syn mice. In addition, PD03 immunotherapy resulted in increased binding of IgGs in the brains of immunized PLP-α-syn mice, indicating that the PD03-induced antibodies entered the brain and accumulated at the sites of α-syn pathology. The active immunotherapy with PD03 reduced the level of α-syn oligomers and GCI density in the brains of PLP-α-syn mice, but did not affect the level of physiological α-syn monomers, which suggested a specificity of the approach for the pathogenic oligomeric species. Further, the PD03 treatment ameliorated the motor deficits, and rescued dopaminergic neurons in the SNc of the PLP-α-syn model. Intriguingly, the reduction of α-syn oligomeric species after PD03 immunotherapy was associated with a reduction of microglial activation in the PLP-α-syn brain. It has been shown that oligomeric α-syn is a major trigger of microglial activation [42–44]. Therefore, the decreased oligomeric α-syn level by PD03 immunotherapy may have contributed to the reduction of neuroinflammation and the related neuroprotective effects. This finding is consistent with previous reports of reduced microglial activation in PDGF-α-syn mice receiving active anti-α-syn immunization [38].

In addition, the ELISA-based competition assay suggested that the PD03-elicited antibodies preferentially bind to the high-molecular-weight α-syn species. This was further supported by the experiment in which the oligomer modulation by Anle138b was applied. We showed that the combined treatment with PD03 and Anle138b resulted in higher plasma titers of free anti-α-syn antibodies (but not anti-KLH antibodies), presumably due to the lower engagement of the anti-α-syn antibodies in the brain because of the modification/lowering of the oligomeric species of α-syn by Anle138b [12]. Indeed, we identified reduced IgG binding in the brains of PLP-α-syn mice receiving combined PD03 + Anle138b therapy as compared to those receiving single PD03 treatment. Taken together, our results support the selective binding of PD03-triggered antibodies to oligomeric α-syn. Importantly, the combined treatment in this experiment did not interfere with the beneficial effects of the single therapies (PD03 or Anle138b alone), suggesting that a critical mass of pathological α-syn can be reduced by the combined therapy as well as the single therapies. As a complete rescue was already achieved with single therapies (PD03 or Anle138b alone), the experimental design did not examine the potential synergistic therapeutic effects.

## Conclusions

The interest in AFFITOPEs® is based on the fact that both PD01 and PD03 have been tested in Phase I clinical trials, and have been shown to be safe and well-tolerated when followed up for over 36 weeks in patients (www.sympath-project.eu). Anle138b is also under testing in a Phase I clinical trial. Our experimental approach demonstrated the efficacy of PD03 and Anle138b in a model of MSA-like α-synucleinopathy and supports further clinical development of these therapies.

## Data Availability

All data generated or analyzed during this study are included in this article.

## Abbreviations

α-syn: α-synuclein
PD: Parkinson’s disease
DLB: Dementia with Lewy bodies
MSA: Multiple system atrophy
PLP: Proteolipid protein
SNc: Substantia nigra pars compacta
SDS: Sodium dodecyl sulfate
GCIs: Glial cytoplasmic inclusions
